# Provider Determinants of Maternal Influenza and Pertussis Vaccination Uptake in South Australia in a Tertiary Healthcare Setting

**DOI:** 10.3390/jcm14030890

**Published:** 2025-01-29

**Authors:** Hassen Mohammed, Kathryn Riley, Michelle Clarke, Mary S. Walker, Helen S. Marshall

**Affiliations:** 1 Women’s and Children’s Health Network, North Adelaide, SA 5006, Australia; 2 The Robinson Research Institute, Adelaide Medical School, The University of Adelaide, Adelaide, SA 5005, Australia

**Keywords:** influenza, pertussis, maternal vaccination, antenatal healthcare, pregnancy

## Abstract

**Background**: In Australia, maternal influenza and pertussis vaccinations have been recommended for every pregnancy since 2010 and 2015, respectively. Aims: This study aimed to determine maternal influenza and pertussis vaccine uptake in South Australia and assess factors associated with vaccine uptake among pregnant women. **Methods**: This retrospective cohort study collected data from the South Australian Pregnancy Record (SAPR) or other medical records of women who delivered at the Women’s and Children’s Hospital from 2016 to 2018. **Results**: Of 2230 complete records, 53.5% received influenza vaccination and 66.5% pertussis vaccination. Maternal vaccine uptake significantly increased from 2016 to 2018: influenza 43.1–61.6%; pertussis 58.7–71.6%. Healthcare provider discussions with pregnant women about maternal vaccines more than doubled the likelihood of influenza (AOR 2.74, 95% CI: 2.21–3.39) and pertussis vaccine uptake (AOR 2.22, 95% CI: 1.77–2.78). Lower vaccine uptake was observed among women attending midwifery clinics (influenza: AOR 0.72, 95% CI: 0.58–0.90; pertussis: AOR 0.67, 95% CI: 0.54–0.84) or private maternity care (influenza: AOR 0.51, 95% CI: 0.34–0.77; pertussis: AOR 0.40, 95% CI: 0.27–0.60). Shared antenatal care increased the uptake of influenza (AOR 1.51, 95% CI: 1.12–2.04) and pertussis (AOR 1.39, 95% CI: 1.00–1.91). Additional adjustment for SAPR versions did not appreciably change the results, although attending private practice was no longer significantly associated with lower vaccine uptake. **Conclusions**: Maternal vaccine uptake varies depending on the antenatal care provider. This study identifies opportunities to improve vaccination access during pregnancy and emphasizes the need for targeted strategies to address provider-related barriers.

## 1. Introduction

Infants aged less than six months and pregnant women are at high risk of severe complications from influenza, including death [1]. Maternal influenza vaccination can significantly mitigate these risks during pregnancy, the early postpartum period, and infancy, particularly for infants under six months who are ineligible for vaccination, providing critical protection during this vulnerable period [2]. While pertussis is less prevalent than influenza, the morbidity and mortality associated with pertussis infections in early infancy remain substantial [3,4]. This risk is especially significant during the first three months of life, before infants complete their primary vaccination series at six months [5].

Maternal vaccination has proven to be an effective strategy to protect the mother while also providing passive antibodies to the newborn through the transfer of transplacental antibodies [6] and breastfeeding [7]. This offers protection during early infancy and reduces the morbidity and mortality associated with vaccine-preventable infections [8,9]. Several countries, including Australia, have integrated influenza and pertussis immunization into their standard antenatal care recommendations [10].

State-funded pertussis vaccination programs for pregnant women were introduced in 2014–2015 in all Australian states and territories [11]. Across all jurisdictions, pregnant women in their third trimester received pertussis vaccines through a wide range of health service providers, supported by a promotional campaign [12]. Maternal pertussis vaccines were incorporated into the National Immunization Program (NIP) in 2018, while influenza vaccine has been funded for all pregnant women through the NIP since 2010 [11]. In April 2015, the Women’s and Children’s Hospital (WCH), the primary tertiary maternity hospital for complex care in South Australia, commenced a maternal immunization program including antenatal health care provider’s recommendation about the two recommended vaccines to pregnant women, the recording of maternal immunizations within the pregnancy hand-held record, and a designated funded registered nurse position within the hospital for delivering antenatal influenza and pertussis vaccination to pregnant women. In Australia, maternity care is provided through public hospitals, private obstetric care, and shared care models involving general practitioners (GPs). Midwife-led care, commonly offered in public hospitals, focuses on continuity of care by midwives throughout pregnancy, while private obstetric care is predominantly managed by obstetricians. Shared care integrates GP services with hospital-based antenatal care. These care models may influence opportunities for vaccination discussions and delivery during pregnancy. This study uniquely examines how the type of maternity care model and provider–patient interactions influence maternal vaccine uptake, addressing a gap in the existing literature. This study uniquely examines how the type of maternity care model and provider–patient interactions influence maternal vaccine uptake, addressing a gap in the existing literature

Despite recommendations, maternal influenza vaccine uptake in Australia was low, with estimates ranging from 10% to 30% [13]. In contrast, maternal pertussis vaccine coverage in 2016 was generally higher, exceeding 70% [13]. Australia currently lacks a cohesive framework for maternal immunization, with no systematic monitoring of maternal vaccine coverage. Monitoring and evaluating the impact of state maternal immunization programs is essential to identifying effective strategies to enhance the uptake of routinely recommended vaccines during pregnancy. This study aimed to estimate maternal influenza and pertussis vaccine uptake in South Australia and examine factors associated with vaccine uptake among pregnant women.

## 2. Materials and Methods

### 2.1. Study Population and Design

This is a retrospective cohort assessing pertussis and influenza vaccination uptake during pregnancy over a consecutive 3-year time frame (2016–2018) to evaluate the antenatal immunisation service delivery at the WCH in South Australia. The WCH is a major tertiary maternity hospital, accounting for around 30% (4800 annual births) of the 16,000 annual births in metropolitan Adelaide in South Australia. At the maternity hospital, most women receive publicly funded care provided by midwives and obstetricians. Additionally, women can opt for shared care between the hospital and their general practitioner. A smaller proportion of births occur among women under privately funded obstetric care. The Immunization Clinic at WCH offers vaccination services to pregnant women, including both inpatients and outpatients.

### 2.2. Inclusion and Exclusion Criteria

Women who gave birth at the WCH in August and September each year (2016–2018) were eligible to be included. Women who delivered outside the study period (2016–2018) or outside the months of August and September, as well as those with missing or incomplete case note data, were excluded from the analysis.

### 2.3. Data Collection Methods and Sources

Specific data pertaining to the uptake of influenza and pertussis vaccination during pregnancy were extracted via medical case notes or from the South Australian Pregnancy Record (SAPR). SAPRs serve as the primary medical record for pregnancy care in South Australia, regularly reviewed and updated during antenatal appointments. Data were recorded in a REDCap [14] online database. Information extracted included confirmation of influenza and pertussis vaccination during pregnancy, date of vaccinations, declined vaccinations, acknowledgment of ticked ‘check box’ that the influenza vaccine and pertussis vaccine had been discussed by a healthcare provider, date of delivery, number of weeks gestation at delivery, and type of antenatal model of care and medical/obstetric history.

### 2.4. Main Outcome Measures

The primary outcome was the proportion of pregnant women delivering newborns during August and September who received the influenza and pertussis vaccination during pregnancy over the 3-year period (2016–2018). The secondary outcome measures were potential factors influencing the uptake of influenza and pertussis vaccination among pregnant women. Explanatory variables that were known potential factors associated with maternal vaccination uptake (i.e., HCP discussion/recommendation, type of antenatal care provider, first antenatal care visit, gestational diabetes, asthma, cardiovascular disease, month and year of delivery) were selected based on findings of previous systematic reviews [15,16] and availability in the medical case notes.

### 2.5. Statistical Analysis

No pre-specified sample size calculation was undertaken as data were retrospectively collected. However, the data collection periods were selected to ensure there would be approximately 800 births during a time period (August and September) allowing for adequate opportunity for maternal vaccination receipt, generating a total sample size of approximately 2400 women over the three-year period (2016–2018). Descriptive analysis, such as proportions for categorical variables and mean (median) for continuous variables, were calculated. The proportion of women receiving influenza and pertussis vaccination during pregnancy was calculated and compared by year using chi square tests. Univariate and multivariable logistic regression were used to estimate the crude and adjusted odds ratios (AOR) and their corresponding 95% confidence intervals to determine factors associated with the uptake of influenza and pertussis vaccines during pregnancy. In the mutually adjusted models, we included all explanatory variables that were known potential predictors of maternal vaccination uptake based on the published literature [15,16]. Additional adjustments to the SAPR versions were conducted in sensitivity analyses to account for potential discrepancies in data collection. Variations in data entry fields across different SAPR versions may impact the accuracy of maternal vaccine records. As overall missing data were very minimal (0.97%), we used all available data in the analyses without multiple imputation. All statistical analyses were performed using Stata version 15.

### 2.6. Ethical Approval

The study protocol was approved by the Human Research Committee of the Women’s and Children’s Hospital Network (WCHN), Adelaide, Australia (Audit 872A/August 2019–24 August 2016).

## 3. Results

Of the total 2252 medical case notes, 22 women who delivered outside the study period and or had missing data were excluded. Information from the remaining 2230 complete case notes was included (Supplementary Figure S1). Overall, 34.0% (*n* = 758) of the women delivered in 2016, 36.3% (*n* = 810) in 2017, and 29.7% (*n* = 662) in 2018. The mean gestational age at delivery was 38.2 ± 2.6 weeks (Table 1). Approximately 22.8% (*n* = 509) received care from Midwifery Group Practice (MGP) or Midwives Clinic (MWC), 11.0% (n = 245) from shared maternity care, 4.9% (*n* = 110) from a private obstetrician, and 1.3% (n = 29) from Aboriginal and Maternal Infant Care (AMIC) at the WCH. The majority of women initiated their antenatal care at the WCH in the first or second trimester (98.5%, *n* = 2197) (Table 1).

**Figure 1:**
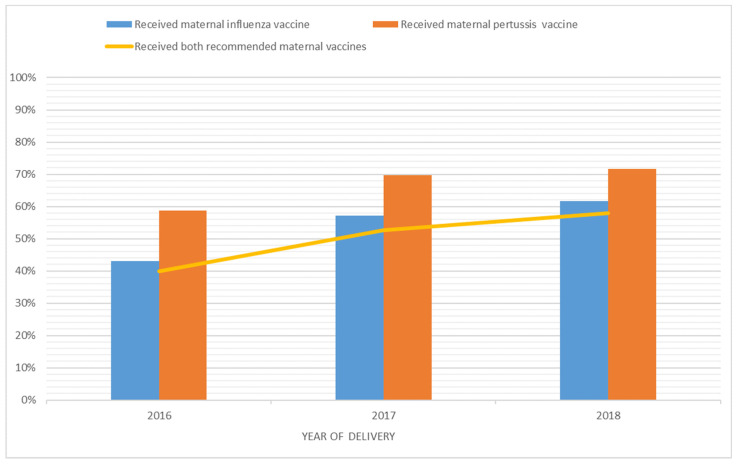
Pertussis and influenza vaccination uptake in pregnant women at the Women’s and Children’s Hospital in South Australia, by birth year, 2016–2018 (N = 2230).

For influenza vaccinations, 28.7% (n = 641) of women had documented evidence of discussions from a healthcare provider compared to 31.7% (n = 706) for pertussis vaccines. Overall, 53.5% (n = 1194) of pregnant women delivering during the study period received an influenza vaccine and 66.5% (n = 1483) received a pertussis vaccine during 2016–2018. Approximately half of the women (49.9%, n = 1113) received both vaccines during pregnancy (Supplementary Table S1). In 2016, 43.1% (n = 327/758) of women received an influenza vaccination during pregnancy, which increased to 56.7% (459/803) in 2017, and increased to 61.6% (408/662) in 2018 (*p* < 0.001). In 2016, 58.7% (445/758) of women received a pertussis vaccination, which increased to 69.6% (n = 564/803) in 2017, and further improvement to 71.6% (474/662) was observed in 2018 (*p* < 0.001) (Figure 1). The majority of vaccinated women with known vaccination dates (n = 1032) received the influenza vaccine in May (32.2%) followed by April (22.6%) and June (20.2%) (Supplementary Table S2). Influenza (50.3% vs. 56.3%) and pertussis vaccine uptake (64.2% vs. 68.5%) were significantly lower for women who delivered in September compared to August (Table 2 and Table 3).

### Predictors of Maternal Influenza and Pertussis Vaccination Uptake

Discussions between healthcare providers and pregnant women about maternal vaccines significantly influenced the uptake of influenza (AOR; 2.74, 95% CI: 2.21–3.39) and pertussis (AOR; 2.22, 95% CI: 1.77–2.78) vaccines during pregnancy. Women who attended MGP or MWC were less likely to receive the influenza (AOR; 0.72, 95% CI: 0.58–0.90) and pertussis vaccines (AOR; 0.67, 95% CI: 0.54–0.84). A similar decline in the uptake of influenza (AOR; 0.51, 95% CI: 0.34–0.77) and pertussis (AOR; 0.40, 95% CI: 0.27–0.60) vaccines was observed in women who attended the majority of their antenatal care at private obstetrician practices compared to those who attended public or shared care maternity services. However, this finding needs to be interpreted cautiously due to the non-mandatory use of the SAPR by private antenatal providers during the study period and, therefore, the potential for vaccination to have occurred but not be recorded in publicly available medical records. Upon additional adjustment for the SAPR versions in the final model, this association was no longer significant (influenza: AOR; 0.91, 95% CI: 0.57–1.45 and pertussis: AOR; 0.80, 95% CI: 0.50–1.27) (Table 2 and Table 3).

The likelihood of receiving both influenza (AOR; 1.51; 95% CI: 1.12–2.04) and pertussis vaccines (AOR; 1.39, 95% CI 1.00–1.91) during pregnancy was significantly higher among women who had attended shared antenatal care through their general practitioner (GP) compared to those who did not. However, after an additional adjustment to SAPR, this association no longer held for the pertussis vaccine. Women who commenced their antenatal care at the WCH after the second trimester were less likely to receive the influenza (AOR; 0.39, 95% CI 0.18–0.84) and pertussis vaccines (AOR; 0.36, 95% CI 0.17–0.74). Women with gestational diabetes had higher odds of receiving both influenza (AOR; 1.33; 95% CI 1.03–1.71) and pertussis vaccines (AOR 1.41; 95% CI 1.07–1.85). However, the presence of other medical conditions associated with increased risk of severe influenza outcomes, such as asthma or cardiovascular diseases, did not influence the receipt of either vaccine (Table 2 and Table 3).

## 4. Discussion

Uptake of influenza vaccination during pregnancy in South Australia significantly improved from 43% in 2016 to 62% in 2018. Recent population-based cohort studies in Australia reported maternal influenza vaccination rates ranging from 35% to 40% [13]. Similarly, in South Australia, maternal pertussis vaccination increased from 59% in 2016 to 72% in 2018, exceeding uptake rates reported in other high-income countries such as the United States (54% in 2018) [17] and the United Kingdom (68% in 2018) [18]. This improvement coincides with the introduction of state-funded antenatal pertussis immunization programs in 2015 and the vaccine’s inclusion in the National Immunization Program (NIP) in 2018. Global variations in maternal vaccination uptake may be influenced by differences in antenatal care models and the integration of vaccination services. Countries with well-integrated vaccination programs as part of routine antenatal care, such as the United Kingdom, tend to achieve higher uptake rates. This highlights the importance of embedding vaccination delivery within antenatal care systems to ensure accessibility and continuity of care, enabling antenatal healthcare providers to play a central role in promoting maternal vaccination uptake globally.

Between 2016 and 2018, a high uptake of influenza vaccination was observed in the autumn months of April and May, indicating increased awareness and availability of the vaccine to provide protection before the typical influenza season (May to October) in Australia. However, there was a lower uptake of the vaccine among women who delivered in September compared to those who delivered in August. This trend may have serious implications for pregnant women, who are at high risk of complications from influenza, particularly during unpredictable influenza seasons.

During the 2018–2019 influenza season, South Australia experienced a surge in influenza notifications earlier than most Australian states [11]. This implies that influenza can severely affect pregnant women year-round, highlighting the need for healthcare providers to offer vaccinations at any stage of pregnancy, irrespective of the season. This is particularly important for pregnant women with comorbidities who are more likely to require hospitalization following influenza [19]. Although our study indicates that women with gestational diabetes mellitus were more likely to receive the recommended maternal vaccinations, this trend was not observed among women with asthma or cardiovascular diseases, despite their higher risk of complications from influenza. This highlights the need for healthcare providers to play an important role in the vaccination decision-making process among pregnant women presenting with comorbidities.

Receiving recommendations from antenatal healthcare provider was a strong predictor of maternal pertussis and influenza vaccination uptake, consistent with previous studies [10]. The present study also suggests that the type of antenatal care provision and access are predictors of vaccination uptake during pregnancy. Our results are also consistent with previous studies [20,21] that showed women who received antenatal care through GP shared care had greater odds of receiving the maternal influenza vaccine, possibly reflecting the expertise of GPs in delivering a range of vaccines or increased opportunities for healthcare provider led vaccination discussions to occur. It is also plausible that shared antenatal care services with frequent GP visits may have provided more opportunities for the delivery of maternal influenza vaccination. Our study found lower maternal vaccine uptake in midwifery-led clinics, highlighting the need for improved vaccine education in MGP and MWC settings. This study did not examine whether this is related to patient or provider factors for those attending MGP or MWC care. However, given the crucial role of midwives, often the most trusted information source for pregnant women, targeted support and maternal vaccination information could significantly enhance vaccine uptake during pregnancy [22]. Addressing system-level or organizational barriers influencing maternal vaccination uptake could assist in improving maternal immunization programs. Barriers in midwifery-led clinics, such as insufficient vaccine training for midwives and resource limitations, may hinder vaccine delivery. Implementing targeted training programs, improving access to vaccines, and integrating automated reminders within antenatal medical records could significantly enhance maternal vaccination uptake [23].

Perinatal data collections in Australian states present an opportunity for data linkage with the Australian Immunization Register (AIR), facilitating accurate assessments of maternal vaccine coverage [22]. Despite AIR having recorded adult vaccination data since 2016, it currently does not incorporate details on pregnancy status. Therefore, a harmonized methodology across all states and territories is needed to integrate such information, thereby facilitating the provision of timely national estimates derived either from jurisdictional data or perinatal data links to the AIR [22].

This study has several limitations. The cohort was drawn from women delivering at a single tertiary maternity hospital, which may limit the generalizability of the findings to the broader population of pregnant women in South Australia. Although maternal vaccination status was verified through SAPR, vaccinations administered in non-traditional settings, such as pharmacies or workplaces where providers may lack access to medical records, were likely underrepresented, potentially leading to an underestimation of vaccine uptake. Furthermore, incomplete documentation of vaccinations in medical records may have contributed to this underestimation; however, the impact of missing data is likely minimal, as only 0.97% of data were missing (n = 22). The study also did not collect information on socio-demographic factors such as maternal age, ethnicity, country of birth, or primary language, which may play a role in vaccine uptake during pregnancy.

In conclusion, antenatal vaccine uptake varies across maternity care models, highlighting opportunities to improve access and vaccination during pregnancy. While uptake of routinely recommended maternal influenza and pertussis vaccines improved between 2016 and 2018, there is still room for improvement. The lack of routine systems to monitor vaccine coverage among pregnant women makes it difficult to track uptake and evaluate the effectiveness of strategies aimed at increasing maternal vaccination rates. A national systematic approach to monitor maternal influenza and pertussis vaccination coverage is urgently needed.

## Figures and Tables

**Table 1:** Characteristics of women reviewed in the maternal vaccination audit in South Australia, 2016–2018.

Characteristic	Study Population (N = 2230) *n* (%)
HCP discussed maternal influenza vaccination (noted within case notes or SAPR)	
No	1589 (71.3%)
Yes	641 (28.7%)
HCP discussed maternal pertussis vaccination (noted within case notes or SAPR)	
No	1524 (68.3%)
Yes	706 (31.7%)
Private care	
No	2120 (95.1%)
Yes	110 (4.9%)
Attended Midwifery Group Practice (MGP) or Midwives Clinic (MWC)	
No	1721 (77.2%)
Yes	509 (22.8%)
Shared maternity care	
No	1985 (89.0%)
Yes	245 (11.0%)
Attended Aboriginal and Maternal Infant Care (AMIC)	
No	2201 (98.7%)
Yes	29 (1.3%)
Late entry to antenatal care (>28 gestational weeks)	
No	2197 (98.5%)
Yes	33 (1.5%)
Gestational diabetes	
No	1917 (86.0%)
Yes	313 (14.0%)
Asthma	
No	2069 (92.8%)
Yes	161 (7.2%)
Cardiovascular disease	
No	2210 (99.1%)
Yes	20 (0.9%)
Chronic kidney disease	
No	2218 (99.5%)
Yes	12 (0.5%)
Month of delivery	
August	1193 (53.5%)
September	1037 (46.5%)
Year of delivery	
2016	758 (34.0%)
2017	810 (36.3%)
2018	662 (29.7%)
Gestational age at delivery, mean (SD)	38.2 ± 2.6

SD, standard deviations; HCP, healthcare provider; SAPR, the South Australian Pregnancy Record. Data are presented as n (%) or mean (SD).

**Table 2:** Factors associated with the uptake of influenza vaccination during pregnancy.

Variable	Received Maternal Influenza Vaccine n/N (%) 1194/2230 (53.5%)	Odds Ratio (OR) (95% CI)	*p*-Value	Adjusted aOR ^a^ (95% CI)	*p*-Value	Additionally Adjusted to SAPR (95% CI)	*p*-Value
HCP discussed maternal influenza vaccination (noted within case notes or SAPR)							
No	754/1589 (47.5%)	Reference		Reference		Reference	
Yes	440/641 (68.6%)	2.42 (1.99, 2.94)	<0.001	2.74 (2.21, 3.39)	<0.001	2.57 (2.06, 3.20)	<0.001
Attended Midwifery Group Practice (MGP) or Midwives Clinic (MWC)							
No	949/1721 (55.1%)	Reference		Reference		Reference	
Yes	245/509 (48.1%)	0.75 (0.61, 0.92)	0.005	0.72 (0.58, 0.90)	0.004	0.66 (0.53, 0.82)	<0.001
Attended private maternity care							
No	1155/2120 (54.5%)	Reference		Reference		Reference	
Yes	39/110 (35.5%)	0.45 (0.30, 0.68)	<0.001	0.51 (0.34. 0.77)	0.001	0.91 (0.58, 1.45)	0.720
Shared maternity care							
No	1037/1985 (52.2%)	Reference		Reference		Reference	
Yes	157/245 (64.1%)	1.63 (1.23, 2.14)	0.001	1.51 (1.12, 2.04)	0.007	1.42 (1.05, 1.93)	0.023
Attended Aboriginal and Maternal Infant Care (AMIC)							
No	1181/2201 (53.7%)	Reference		Reference		Reference	
Yes	13/28 (44.8%)	0.70 (0.33, 1.46)	0.346	0.72 (0.34, 1.50)	0.388	0.66 (0.32, 1.37)	0.272
Late entry to antenatal care (>28 gestational weeks)							
No	1184/2197 (53.9%)	Reference		Reference		Reference	
Yes	10/33 (30.3%)	0.37 (0.17, 0.78)	0.009	0.39 (0.18, 0.84)	0.017	0.38 (0.17, 0.85)	0.020
Gestational diabetes							
No	1009/1917 (52.6%)	Reference		Reference		Reference	
Yes	185/313 (59.1%)	1.30 (1.02, 1.65)	0.034	1.33 (1.03, 1.71)	0.026	1.27 (0.99, 1.63)	0.059
Asthma							
No	1113/2069 (53.8%)	Reference		Reference		Reference	
Yes	81/161 (50.3%)	0.86 (0.63, 1.19)	0.394	0.87 (0.62, 1.22)	0.437	0.82 (0.58, 1.16)	0.285
Cardiovascular disease							
No	1181/2220 (53.4%)	Reference		Reference		Reference	
Yes	13/20 (65.0%)	1.61 (0.64, 4.07)	0.307	1.65 (0.55, 4.91)	0.368	1.43 (0.49, 4.18)	0.504
Chronic kidney disease							
No	1188/2218 (53.6%)	Reference		References		References	
Yes	6/12 (50.0%)	0.86 (0.27, 2.69)	0.805	0.61 (0.21, 1.74)	0.357	0.68 (0.20, 1.30)	0.538
Month of delivery							
August	672/1193 (56.3%)	Reference		Reference		Reference	
September	522/1037 (50.3%)	0.78 (0.66, 0.92)	0.005	0.62 (0.52, 0.75)	<0.001	0.64 (0.53, 0.77)	<0.001
Year of delivery							
2016	327/758 (43.1%)	Reference	<0.001 ^#^	Reference	<0.001 ^#^	Reference	<0.001 ^#^
2017	459/803 (56.7%)	1.72 (1.41, 2.10)	<0.001	1.89 (1.53, 2.35)	<0.001	1.74 (1.39, 2.18)	<0.001
2018	408/662 (61.6%)	2.11 (1.71, 2.61)	<0.001	1.91 (1.53, 2.39)	<0.001	2.00 (1.58, 2.52)	<0.001
SAPR version							
Latest version	915/1594 (57.4%)	Reference	<0.001 ^#^			Reference	<0.001 ^#^
Older version	251/461 (54.5%)	0.88 (0.72, 1.09)	0.259			0.78 (0.61, 0.99)	0.041
Partial	11/41 (26.8%)	0.27 (0.13, 0.54)	<0.001			0.25 (0.12, 0.53)	<0.001
No SAPR	17/133 (12.8%)	0.10 (0.06, 0.18)	<0.001			0.12 (0.07,0.21)	<0.001

CI, confidence interval; HCP, healthcare provider; SAPR, the South Australian Pregnancy Record. ^a^: Mutually adjusted. ^#^ Global *p*-value.

**Table 3:** Factors associated with the uptake of the pertussis vaccination during pregnancy.

Variable	Received Maternal Pertussis Vaccine n/N (%) 1483/2230 (66.5%)	Odds Ratio (OR) (95% CI)	*p*-Value	Adjusted aOR ^a^ (95% CI)	*p*-Value	Additionally Adjusted to SAPR (95% CI)	*p*-Value
HCP discussed maternal influenza vaccination (noted within case notes or SAPR)							
No	945/1524 (62.0%)	Reference		Reference		Reference	
Yes	538/706 (76.2%)	1.96 (1.60, 2.40)	<0.001	2.22 (1.77, 2.78)	<0.001	2.03 (1.61, 2.57)	<0.001
Attended Midwifery Group Practice (MGP) or Midwives Clinic (MWC)							
No	1174/1721 (68.2%)	Reference		Reference		Reference	
Yes	309/509 (60.7%)	0.71 (0.58, 0.88)	0.002	0.67 (0.54, 0.84)	0.001	0.60 (0.48, 0.75)	<0.001
Attended private maternity care							
No	1432/2120 (67.6%)	Reference		Reference		Reference	
Yes	51/110 (46.4%)	0.41 (0.28, 0.61)	<0.001	0.40 (0.27, 0.60)	<0.001	0.80 (0.50, 1.27)	0.359
Shared maternity care							
No	1300/1985 (65.5%)	Reference		Reference		Reference	
Yes	183/245 (74.7%)	1.55 (1.14, 2.10)	0.004	1.39 (1.00, 1.91)	0.045	1.26 (0.91, 1.74)	0.154
Attended Aboriginal and Maternal Infant Care (AMIC)							
No	1465/2201 (66.6%)	Reference		Reference		Reference	
Yes	18/29 (62.1%)	0.82 (0.38, 1.74)	0.611	0.78 (0.36, 1.70)	0.543	0.69 (0.32, 1.48)	0.347
Late entry to antenatal care (>28 gestational weeks)							
No	1469/2197 (66.9%)	Reference		Reference		Reference	
Yes	14/33 (42.4%)	0.36 (0.18, 0.73)	0.005	0.36 (0.17, 0.74)	0.006	0.37 (0.17, 0.81)	0.013
Gestational diabetes							
No	1256/1917 (65.5%)	Reference		Reference		Reference	
Yes	227/313 (72.5%)	1.38 (1.06, 1.81)	0.015	1.41 (1.07, 1.85)	0.013	1.32 (1.01, 1.74)	0.042
Asthma							
No	1378/2069 (66.6%)	Reference		Reference		Reference	
Yes	105/161 (65.2%)	0.94 (0.67, 1.31)	0.720	0.95 (0.67, 1.36)	0.811	0.90 (0.62, 1.29)	0.577
Cardiovascular disease							
No	1470/2210 (66.5%)	Reference		Reference		Reference	
Yes	13/20 (65.0%)	0.93 (0.37, 2.35)	0.886	0.94 (0.35, 2.52)	0.910	0.79 (0.29, 2.08)	0.636
Month of delivery							
August	817/1193 (68.5%)	Reference		Reference		Reference	
September	666/1037 (64.2%)	0.82 (0.69, 0.98)	0.034	0.68 (0.56, 0.83)	<0.001	0.72 (0.59, 0.88)	0.001
Year of delivery							
2016	445/758 (58.7%)	Reference	<0.001 ^#^	Reference	<0.001 ^#^	Reference	<0.001 ^#^
2017	564/810 (69.6%)	1.61 (1.30, 1.98)	<0.001	1.68 (1.34, 2.09)	<0.001	1.54 (1.22, 1.95)	<0.001
2018	474/662 (71.6%)	1.77 (1.41, 2.21)	<0.001	1.57 (1.24, 1.97)	<0.001	1.61 (1.27, 2.04)	<0.001
SAPR version							
Latest version	1222/1594 (70.4%)	Reference	<0.001 ^#^			Reference	<0.001 ^#^
Older version	315/461 (68.3%)	0.90 (0.72, 1.13)	0.396			0.84 (0.66, 1.08)	0.183
Partial	19/41 (46.3%)	0.36 (0.19, 0.67)	0.001			0.33 (0.17, 0.65)	0.001
No SAPR	26/133 (19.6%)	0.10 (0.06, 0.15)	<0.001			0.11 (0.07, 0.18)	<0.001

CI, confidence interval; HCP, healthcare provider; SAPR, the South Australian Pregnancy Record. ^a^: Mutually adjusted. ^#^ Global *p*-value.

## Data Availability

Data can be made available upon reasonable request to the corresponding author.

## Supplementary Materials

The following supporting information can be downloaded at: https://www.mdpi.com/article/10.3390/jcm14030890/s1, Figure S1: Flow Chart. Table S1. Annual trends in maternal vaccination uptake and healthcare provider (HCP) recommendations (2016–2018). Table S2. Monthly receipt of influenza vaccine during pregnancy for women who delivered at the Women’s and Children’s Hospital in South Australia, from August to September, between 2016 and 2018 (N = 1189).
