# Supplementary material for: Provider Determinants of Maternal Influenza and Pertussis Vaccination Uptake in South Australia in a Tertiary Healthcare Setting
Source: J Clin Med. 2025 Jan 29;14(3):890. doi: 10.3390/jcm14030890 (PMC11818772; doi:10.3390/jcm14030890)
Supplement: Supplementary file 1 [file jcm-14-00890-s001.zip › Supplementary Tables.pdf]

Supplementary Table S1. Annual trends in maternal vaccination uptake and healthcare provider (HCP) recommendations (2016-2018)

	2016-2018 (n=2230)	2016 (n=758)	2017 (n=810)	2018 (n=662)	*P-value*
Received maternal influenza vaccine	1194 (53.5 %)	327 (43.1%)	459 (56.7%)	408 (61.6%)	< 0.001
Received maternal pertussis vaccine	1483(66.5%)	445 (58.7%)	564 (69.6%)	474 (71.6%)	< 0.001
Received both vaccines	1113 (49.9%)	302/ (39.8%)	427 (52.7 %)	384 (58.0%)	< 0.001
HCPs discussed maternal influenza vaccination	641 (28.7%)	214 (28.2%)	154 (19.0%)	273 (41.2%)	< 0.001
HCPs discussed maternal pertussis vaccination	706 (31.7%)	239 (31.5%)	170 (21.0%)	297 (44.9%)	< 0.001
HCPs discussed both maternal vaccination	491 (22.0%)	166 (21.9%)	118 (14.6%)	207 (31.3%)	< 0.001

Supplementary Table S2. Monthly receipt of influenza vaccine during pregnancy for women who delivered at the [Hospital Name Removed] in South Australia, from August to September, between 2016 and 2018 (N=1189)

**Month**	**Frequency (%)**
January	3 (0.3%)
February	4 (0.3%)
March	22 (1.8%)
April	233 (19.5%)
May	332 (27.8%)
June	208 (17.4%)
July	161 (13.5%)
August	60 (5.0%)
September	8 (0.7%)
December	1 (0.1%)
Unknown vaccination date	162 (13.6%)
**Total**	1194 (100.0)
