# Supplementary figures and images for: Provider Determinants of Maternal Influenza and Pertussis Vaccination Uptake in South Australia in a Tertiary Healthcare Setting
Source: J Clin Med. 2025 Jan 29;14(3):890. doi: 10.3390/jcm14030890 (PMC11818772; doi:10.3390/jcm14030890)

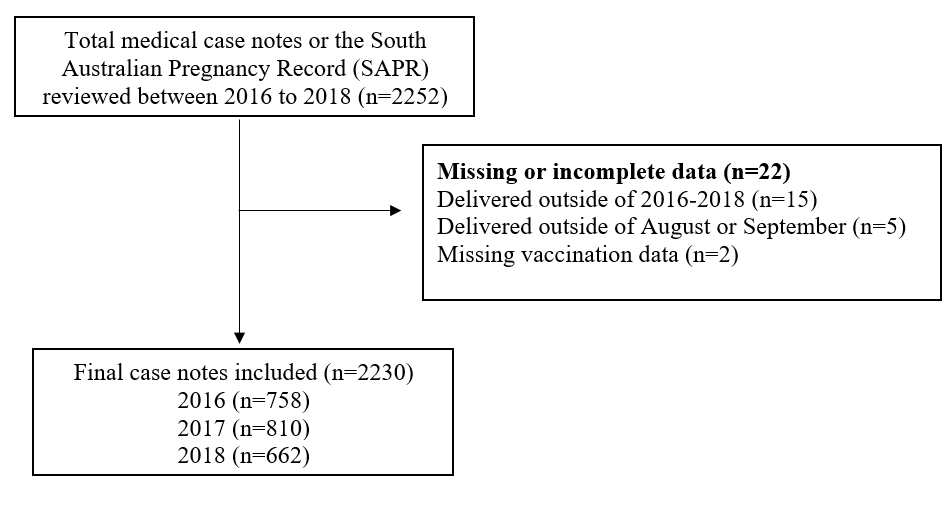

Supplement: Supplementary file 1 [file jcm-14-00890-s001.zip › Supplementary Figure 1.PNG]
